# Complementary analysis of proteome‐wide proteomics reveals changes in RNA binding protein‐profiles during prostate cancer progression

**DOI:** 10.1002/cnr2.1886

**Published:** 2023-08-17

**Authors:** Erika Aikio, Sonja Koivukoski, Elina Kallio, Nithin Sadeesh, Einari A. Niskanen, Leena Latonen

**Affiliations:** ^1^ Institute of Biomedicine University of Eastern Finland Kuopio Finland; ^2^ Foundation for the Finnish Cancer Institute Helsinki Finland

**Keywords:** castration resistance, metastasis, prostate cancer, RNA binding proteins

## Abstract

**Background:**

Accumulating evidence indicates importance of RNA regulation in cancer. This includes events such as splicing, translation, and regulation of noncoding RNAs, functions which are governed by RNA binding proteins (RBPs).

**Aims:**

To find which RBPs could be relevant for prostate cancer, we performed systematic screening of RBP expression in clinical prostate cancer.

**Methods and Results:**

We interrogated four proteome‐wide proteomics datasets including tumor samples of primary, castration resistant, and metastatic prostate cancer. We found that, while the majority of RBPs are expressed but not significantly altered during prostate cancer development and progression, expression of several RBPs increases in advanced disease. Interestingly, most of the differentially expressed RBPs are not targets of differential posttranscriptional phosphorylation during disease progression. The RBPs undergoing expression changes have functions in, especially, poly(A)‐RNA binding, nucleocytoplasmic transport, and cellular stress responses, suggesting that these may play a role in formation of castration resistance. Pathway analyzes indicate that increased ribosome production and chromatin‐related functions of RBPs are also linked to castration resistant and metastatic prostate cancers. We selected a group of differentially expressed RBPs and studied their role in cultured prostate cancer cells. With siRNA screens, several of these were indicated in survival (DDX6, EIF4A3, PABPN1), growth (e.g., EIF5A, HNRNPH2, LRRC47, and NVL), and migration (e.g., NOL3 and SLTM) of prostate cancer cells. Our analyzes further show that RRP9, a U3 small nucleolar protein essential for ribosome formation, undergoes changes at protein level during metastasis in prostate cancer.

**Conclusion:**

In this work, we recognized significant molecular alterations in RBP profiles during development and evolution of prostate cancer. Our study further indicates several functionally significant RBPs warranting further investigation for their functions and possible targetability in prostate cancer.

## 1 INTRODUCTION

Prostate cancer is the most common cancer in men in developed countries and one of the leading causes of cancer death.^1^ The use of prostate specific antigen (PSA) for screening of asymptomatic men has reduced disease‐specific mortality of prostate cancer, but screening is associated with overdiagnosis.^2^ For localized prostate cancer, prostatectomy and radiation therapy are generally effective treatment modalities. For advanced prostate cancer, androgen deprivation therapy (ADT) is the primary and initially effective treatment due to that prostate cancer is driven by androgens and the androgen receptor (AR). ADT will, however, eventually lead to formation of resistance and the emergence of so‐called castration‐resistant prostate cancer (CRPC).^3^ CRPC is highly aggressive and has poor prognosis while, currently, no curative forms of treatment exist for metastatic CRPC (mCRPC).^3^, ^4^ Hence, novel therapeutic targets and treatment options for prostate cancer are required, warranting better understanding of molecular mechanisms of especially disease progression from primary prostate cancer to CRPC.

Research during the last decades has revealed the landscape of genetic alterations and the resulting changes in gene expression in prostate cancer due to rapid advancements in sequencing technologies.^4^, ^5^ Yet, the accompanying altered cellular functions and routes to them are not completely understood. Recent advancements in quantitative proteomics methods have enabled assessment of proteins in a large scale from normal and tumor tissues.^6^, ^7^, ^8^ These studies have the potential to reveal molecular alterations in cancer that have previously gone unidentified, gaining valuable information for understanding tumor evolution and possibly relevant for cancer treatments. For example, we recently showed by integrating genome‐wide genetic, transcriptomic, and proteomic data from the same tumors that, compared to sequencing data only, additional altered pathways are found based on proteomics data in prostate cancer.^9^ Similar observations have been reported also in other cancer types.^10^, ^11^, ^12^ These reports indicate that certain frequent and systematic changes in molecular pathways in cancer are detectable mainly at the protein level. This prompted us to investigate further the available proteomics datasets on prostate cancer to identify pathways and groups of proteins that have gained less attention so far, but which are systematically altered based on proteomics studies.^8^

We previously found that several cellular functions governed by RNA binding proteins (RBPs), including splicing and translation, are often altered in prostate cancer, particularly in advanced tumors.^8^, ^9^ Since RBPs are an understudied group of proteins for their functions in cancer, we decided to systematically analyze RBP expression in prostate tumors to recognize RBPs with significant changes in expression in either primary or advanced prostate cancer. Here we find that the expression of RBPs from several functional groups is altered in castration resistant and metastatic prostate cancer, while in primary cancer the systematic alterations in RBP expression or phosphorylation are less frequent. We identify several interesting RBPs to study further for their possible role in prostate cancer development and progression, and we perform functional screens for a group of these to show their relevance in modulating growth, migration, and apoptosis of prostate cancer cells.

## 2 MATERIALS AND METHODS

### 2.1 Combining an RBP master list

First, a list of known RBPs was combined based on the work of Gerstberger et al.^13^ and Sundararaman et al.^14^ Gene symbols were unified using HGNChelper package^15^ and duplicated gene symbols were removed. As a result, an RBP master list of 1784 RBPs was produced and used to extract RBPs from the prostate cancer proteomics datasets.

### 2.2 Proteomics datasets and analysis

Four datasets of prostate cancer proteomics were utilized in this study. The datasets were generated with different mass spectrometry methods and contained proteomics data of different stages of prostate cancer (Table 1). Sinha et al.^16^ dataset contained data from localized, treatment naïve prostate tumor samples with information about their clinical International Society of Urological Pathology (ISUP) group, clinical T category, presence or absence of E‐26 transformation‐specific gene family (ETS) fusion, and biochemical recurrence (BCR). Latonen et al.^9^ dataset included samples of benign prostate hyperplasia (BPH), primary prostate cancer tumors (PC), and localized CRPC. Iglesias‐Gato et al.^17^, ^18^ dataset contained data from nonmalignant (C), localized PC tumor (Loc), and bone metastasis (Met) samples. The bone metastasis samples included hormone naïve (HN) and CRPC samples, and one sample was from a patient that had gone through a short ADT treatment. Drake et al.^19^ dataset contained phosphoproteome data of benign, local PC (LocPC), and metastatic CRPC (MetCRPC) samples including phosphoserine and phosphothreonine peptides.

**TABLE 1:** Prostate cancer proteomics datasets utilized in this study

References	Method	Number of proteins or phosphopeptides*	Sample types (number of samples)
Sinha et al. 2019^16^	Shotgun LC–MS/MS	3397	PC (76)
Latonen et al. 2018^9^	SWATH‐MS	3394	BPH (10), PC (17), CRPC (11)
Iglesias‐Gato et al. 2016; Iglesias‐Gato et al. 2018^17^, ^18^	SILAC LC–MS/MS	9827	C (8), Loc (28), Met: HN, CRPC and short ADT (22)
Drake et al. 2016^19^	Phosphopeptide enrichment + MS/MS	8051*	Benign (5), LocPC (6), MetCRPC (16)

* Number of phosphopeptides.

Preprocessing, heat map visualization, and statistical testing was performed in RStudio (R version 4.0.5), and Venny 2.1 was used for Venn diagrams.^20^ Gene symbols in all datasets were unified using HGNChelper package,^15^ and log2‐transformed data was used for all datasets. Since the datasets had been compiled differently, dataset‐specific preprocessing was performed to retain unique values for each gene. When multiple values per gene were found, unique gene symbols based on the least number of missing values were included in the analysis or preferring reviewed proteins to unreviewed proteins based on Uniprot database. When multiple isoforms per gene were present (for Sinha dataset), the one with highest median expression was retained. To allow hierarchical clustering, further filtering was performed with datasets containing missing values (genes with 57/79 values missing were removed from Sinha dataset, samples having less than 2/3 values in at least one sample group for Iglesias‐Gato dataset). In addition, based on quality control analyzes by evaluation of distributions, principal component analyzes, and correlation heatmaps, two samples from Iglesias‐Gato (Ratio_PC32_Met_HN and Ratio_PC63_C) and Drake (BS86687_cancer and RA53_lt._lung_lt._femur_mean) datasets were excluded from the analysis. Proteomics datasets were visualized with ggplot2^21^ and ComplexHeatmap packages.^22^ For heatmaps, Z scores were used, and samples and proteins in each dataset were hierarchically clustered using Euclidean as a distance calculation method and complete as clustering method.

Differences in RBP expression between sample groups were tested with either ANOVA or Kruskal‐Wallis test. ANOVA was performed to RBPs that fulfilled assumptions of equal variances and normal distribution tested with Levene's test function in car package^23^ and Shapiro–Wilk test, respectively. Benjamini‐Hochberg false discovery rate (FDR) correction was used in correction for multiple testing. RBPs with a *p*‐value <.05 after FDR were considered statistically significant. ANOVA was followed with Tukey post hoc test and Kruskal‐Wallis test with Dunn's test.^24^ In Latonen, Iglesias‐Gato, and Drake datasets, comparisons were performed between the sample groups. For Sinha dataset only including primary tumors, comparisons were performed between clinical categories BCR (Yes vs. No), T category (T1 vs. T2), ETS fusion (True vs. False) and diagnostic ISUP group (1 + 2 vs. 3). Volcano‐plots were generated with ggplot2^21^ and ggrepel^25^ in R using ANOVA and Kruskall‐Wallis posthoc *p*‐values and log2 fold‐changes of average expression values.

### 2.3 Pathway analysis

Pathway analysis was performed with DAVID Bioinformatics Resources 6.8^26^ for statistically significant RBPs against OMIM_DISEASE, GOTERM_BP_FAT, GOTERM_CC_FAT, GOTERM_MF_FAT, KEGG_PATHWAY and REAC‐TOME_PATHWAY resources. Terms and their adjusted *p*‐values were extracted from the results table. Data frames containing terms enriched in early and late‐stage PC were merged and filtered so that each term had at least one of the groups FDR adjusted *p*‐value under 0.05. The 20 annotation clusters with highest enrichment score in each stage were plotted and labeled according to the most common and descriptive functional group.

### 2.4 Cell culture

Cells were obtained from ATCC (Manassas, Virginia, USA). All cells were cultured in RPMI‐1640 (BioWhittaker, Lonza, Basel, Switzerland) with 10% FBS (Gibco, Waltham, Massachusetts, USA), 1% L‐glutamine (Gibco), and 1% Penicillin–Streptomycin (Gibco) and maintained in 37°C with 5% CO2 in humified atmosphere.

### 2.5 siRNA transfections

For each gene, two siRNAs (SilencerTM Select Pre‐Designed siRNA, Ambion, ThermoFischer Scientific, Waltham, Massachusetts, USA) were selected to target different parts of the mRNA (Supplementary Table 1). As a control, Silencer™ Negative Control No. 1 siRNA was used. siRNAs were used at 20 nM concentration and reverse transfected with INTERFERin (Polyplus transfection, Illkirch, France) according to manufacturer's instructions using Opti‐MEM (ThermoFischer Scientific, Waltham, Massachusetts, USA, catalog number 31985062) medium without serum.

### 2.6 Analysis of RNA expression

To confirm siRNA efficiency, RNA was extracted from PC‐3 cells 48 h after transfection using TRI Reagent Solution (Invitrogen, ThermoFischer Scientific) according to manufacturer's protocol. RT‐qPCR using the Maxima SYBR Green/ROX qPCR Master Mix (ThermoFischer Scientific) according to manufacturer's instructions and run using Light Cycler 480 (Roche). TBP3 was used as the housekeeping gene. Statistical significance was assessed with two‐tailed t‐test from cell line experiments and with Mann–Whitney test from previously published expression datasets.

### 2.7 Proliferation assay

For proliferation assay, transfected PC‐3, 22Rv1, and LNCaP cells were plated on 96‐well‐plates in densities of 6000, 7500, and 10 000 cells/well, respectively. Cell growth was monitored using IncuCyte live cell imaging system (Essen BioScience, Sartorius, Göttingen, Germany) for 6 days and the data was processed using IncuCyte 2021C software (Sartorius).

### 2.8 Apoptosis assay

For determining rate of apoptosis, Caspase‐3/7 assay (Essen BioScience) was used. PC‐3, LNCaP, and 22Rv1 cells were reverse transfected in 96‐well‐plates in densities of 10 000, 15 000, and 12 000 cells/well, respectively. The next day, the cells were replenished with fresh medium containing Caspase‐3/7 assay reagent in concentrations 1.25 μM for PC‐3 and 22Rv1 cells, and 0.5 μM for LNCaP cells. Red fluorescence was measured at 72 h and analyzed using adherent cell‐by‐cell analysis with the IncuCyte‐system.

### 2.9 Migration assay

For analyzing migration ability of the cells, PC‐3 cells were transferred to 96‐well Image Lock Microplate (Essen BioScience) with 30 000 cells/well on the day following transfection. The following day, IncuCyte Woundmaker Tool (Essen BioScience) was used to create a wound according to manufacturer's instructions. The plate was imaged at 36 h and analyzed with Scratch Wound Analysis Software Module (Sartorius) to monitor the migration of the cells to the wound area.

## 3 RESULTS

### 3.1 Majority of known RBPs are expressed in clinical prostate cancer

First, we compiled a list of known RBPs based on the work in Gerstberger et al.^13^ and Sundararaman et al.^14^ The protein list in Gerstberger et al.^13^ contained RBPs identified based on them containing an RNA binding domain. The list also included manually added proteins that were previously shown to be involved in RNA binding but left unrecognized in a search for RNA binding domain‐containing proteins. In addition, proteins that were, for example, known DNA binding proteins, products of pseudogenes, or resulting from gene duplications had been removed. Sundararaman et al.^14^ also compiled their RBP list based on proteins containing RNA binding domains, but they only kept RBPs that bind pre‐mRNA or mRNA. In addition, certain RBPs had been added based on existing information of RNA binding in literature. Further, RBPs discovered in Castello et al.^27^ of mRNA interactome in HeLa cells had been included. Gene symbols were used as identifiers for RBPs, and their correctness was checked using HGNChelper package.^16^ Duplicated gene symbols were removed. As a result, a master list of 1784 RBPs was produced and subsequently used to extract RBPs from the prostate cancer proteomics datasets.

**FIGURE 1:**
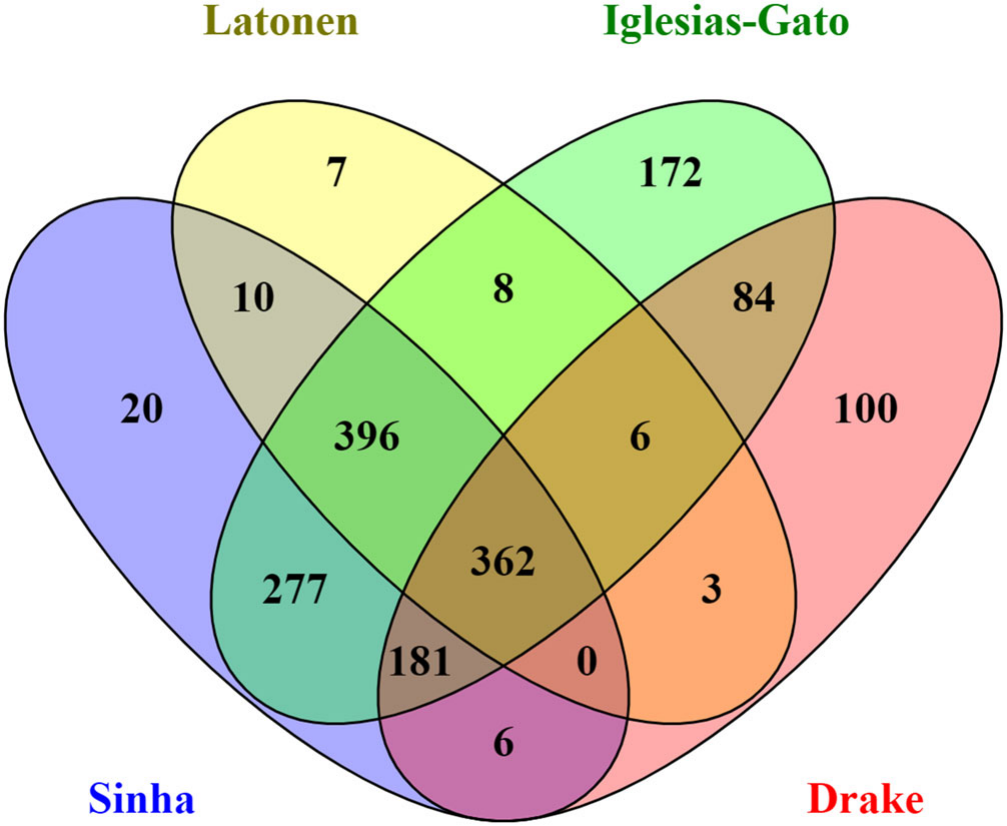
RBPs expressed in prostate cancer. In total, 1651 RBPs were identified as expressed in the normal prostate or prostate cancer samples based on the four proteomics datasets used in the study. Venn diagram shows overlap of identified RBPs between the datasets.

Second, we extracted RBPs were from the four available datasets reporting large‐scale proteomics from clinical prostate cancer samples (Table 1). Sinha et al.^16^ dataset contained data from localized, treatment naïve prostate tumor samples with information about their clinical categories, including T‐stage, diagnostic ISUP group grade, ETS fusion status, and BCR. Latonen et al.^9^ dataset included samples of BPH, primary prostate cancer tumors (PC), and localized castration resistant prostate cancer (CRPC). Iglesias‐Gato et al.^17^, ^18^ dataset contained nonmalignant prostate tissue (C), localized prostate cancer (Loc), and bone metastasis (Met) samples. The Met samples included HN and CRPC samples, and one sample was from a patient that had gone through a short ADT treatment. Drake et al.^19^ dataset contained phosphoproteome data of benign, local prostate cancer (LocPC), and MetCRPC samples including phosphoserine and phosphothreonine peptides. In total, 1651 RBPs were included in one or several of the prostate cancer datasets (Figure 1, Supplementary Table 2), covering over 90% of RBPs in our master list. A noteworthy overlap is evident between the datasets, supporting the idea that a significant proportion of RBPs that are expressed in prostate cancer.

Next, we performed clustering analysis of the RBPs in each dataset. Due to the large number of missing values in the Iglesias‐Gato and Sinha datasets, a filtering step was performed for successful clustering analysis with these datasets. In the Latonen and Iglesias‐Gato datasets, samples cluster mainly, but not exclusively, according to their sample type (benign, primary, or CRPC/Met) (Supplementary Figure 1A,B). In the Sinha dataset, there is no clustering of primary prostate cancer samples based on the clinical categories that were studied, including T‐stage, ISUP group grade, ETS fusion status, or BCR (Supplementary Figure 1C). In the Drake dataset containing phosphopeptide data, there are two major clusters that follow the cancer stage, with the first cluster containing the metastatic CRPC samples and the second cluster containing the benign and LocPC samples (Supplementary Figure 1D). These results indicate that the overall expression of RBPs is somewhat altered based on the disease stage, and that the phosphorylation of RBPs in metastatic CRPC differs from that of benign and local disease.

### 3.2 Expression of most RBPs is homogenous between clinical categories of curable, localized primary PC

**FIGURE 2:**
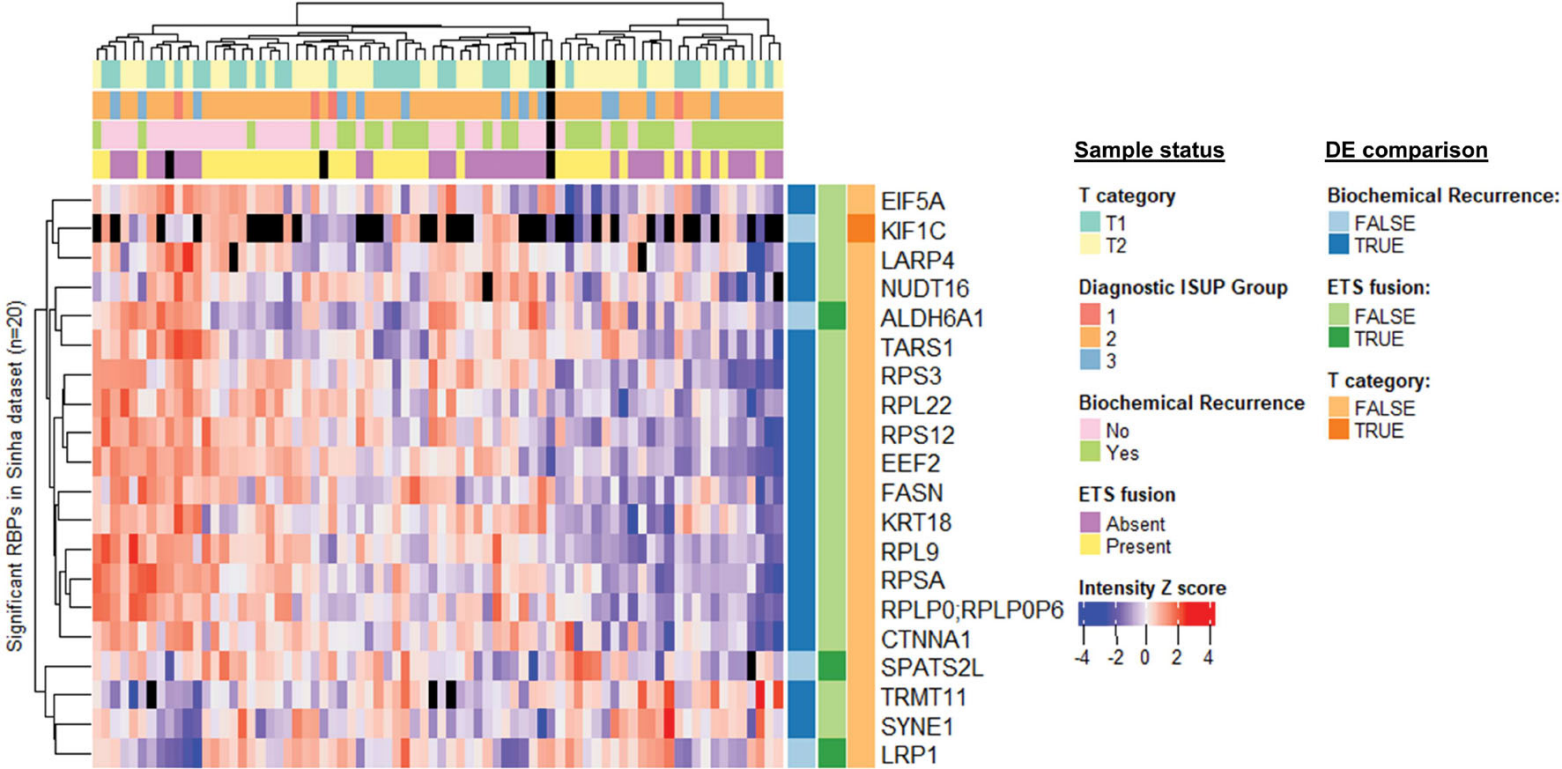
Expression of RBPs is relatively homogenous in primary prostate cancer. Clustering analysis of differentially expressed (DE) RBPs in primary prostate cancer in the Sinha dataset. Only 20 RBPs were differentially expressed in comparisons between samples belonging to T1 or T2 category, with or without BCR during the follow‐up period, or with or without an ETS fusion. Black color indicates a missing value.

The Sinha dataset contains samples from localized primary tumors of curable prostate cancer (PC). The expression of RBPs was compared between the clinical categories (T‐stage, ISUP grade group, and BCR) and between samples positive and negative for ETS gene fusions. No differentially expressed RBPs were found between the available ISUP groups (1 + 2 vs. 3), and only a few RBPs were differentially expressed between any of the other groups (Figure 2). Sixteen RBPs where differentially expressed between patients with recurrence based on elevated PSA in comparison to those without recurrence (BCR). Comparison between samples with or without ETS gene fusion resulted in three differentially expressed RBPs, namely ALDH6A1, SPATS2L, and LRP1. Between T1 and T2 stage tumors, a single RBP, KIF1C, was differentially expressed. Overall, these results suggest that the expression of RBPs in curable, localized primary prostate cancer is relatively homogenous between the different clinical categories and in tumors with different ETS fusion status.

### 3.3 Prostate cancer progression involves significant changes in RBP profiles

**FIGURE 3:**
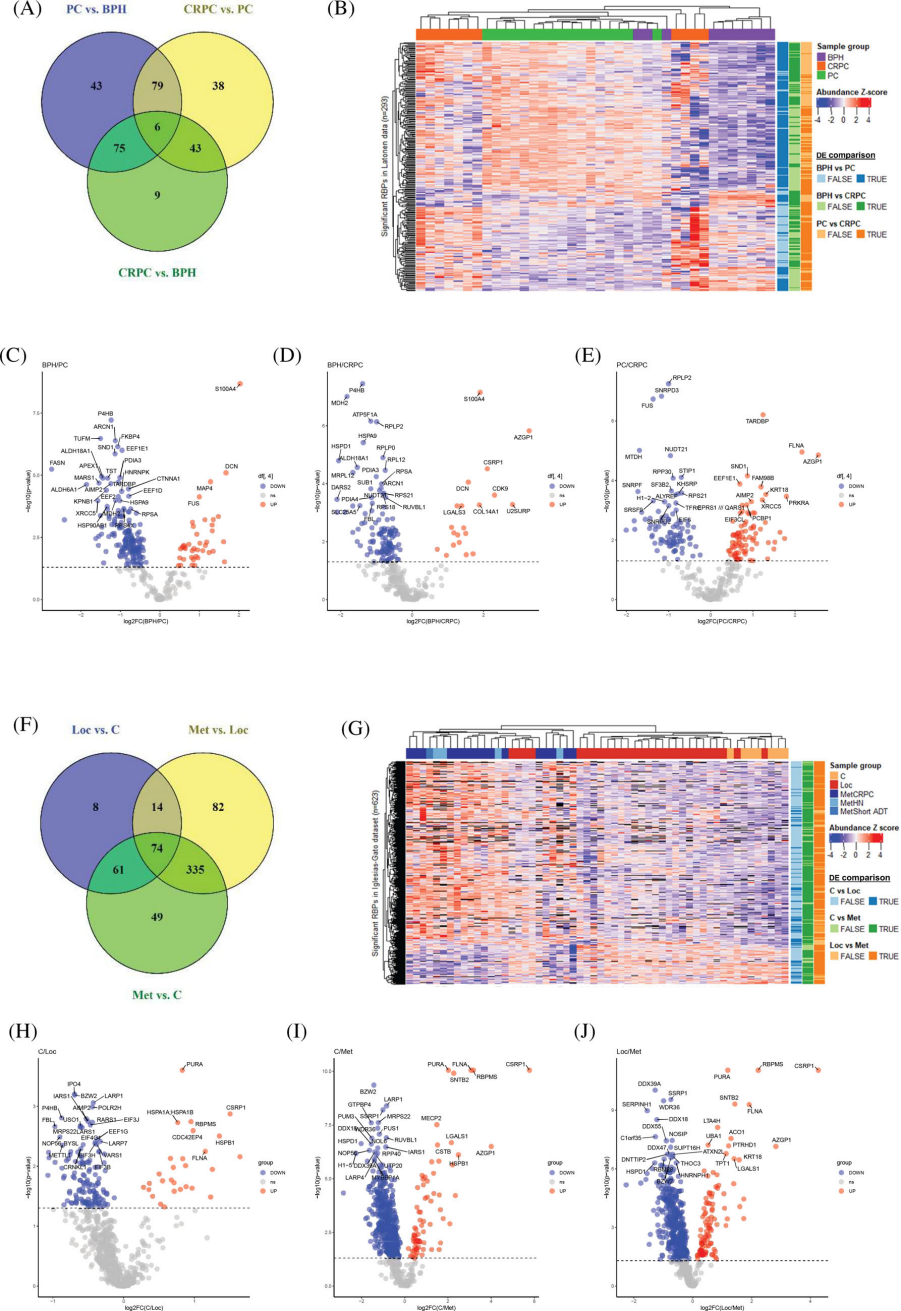
Expression of RBPs differs between primary and advanced prostate cancer. RBPs differentially expressed (DE) between the clinical phases of prostate cancer based on the Latonen dataset (A–E), and the Iglesias‐Gato dataset (F–J). Venn diagrams (A, F), heatmaps of clustering analysis (B, G), and volcano plots with top 30 proteins indicated (C–E, H–J) are shown. Black color indicates a missing value in heatmaps.

To investigate whether the expression of RBPs is altered during progression of prostate cancer, we studied RBP profiles in datasets which contained comparable samples from both primary and advanced tumors. The expression levels of RBPs in BPH, PC, and CRPC samples were compared within the Latonen dataset, and C, Loc, and Met samples within the Iglesias‐Gato dataset. In the Latonen dataset, 293 RBPs were discovered to be differentially expressed (Figure 3A). These differentially expressed RBPs clustered mostly according to the sample groups, which indicates distinctive expression of the RBPs in the different stages of prostate cancer (Figure 3B). The greatest number of differentially expressed RBPs, 203, was found by comparing PC to BPH, with 39 RBPs downregulated and 164 upregulated. For the majority of RBPs in this comparison, the expression was higher in PC compared to BPH. The second highest number of differentially expressed RBPs was discovered in CRPC versus PC comparison with 166 RBPs, with 78 RBPs downregulated and 88 upregulated. There, the expression of approximately half of the RBPs was increased in CRPC compared to PC. In CRPC versus BPH comparison, 133 RBPs were differentially expressed, with expression of most RBPs higher in CRPC compared to BPH (with 20 RBPs downregulated and 113 upregulated). Volcano plots of each comparison is shown in Figure 3C–E.

In the Iglesias‐Gato dataset, 623 RBPs were differentially expressed when comparing C, Loc, and Met stages (Figure 3F). Hierarchical clustering of the samples based on the expression of differentially expressed RBPs shows division into two principal clusters (Figure 3G). The first major cluster contains Met samples and four Loc samples, and the second cluster contains the rest of the Loc samples and all C samples (Figure 3G). While the clustering does not perfectly follow disease stage, the metastatic samples seem to differ from Loc and C samples more than C and Loc samples from each other (Figure 3G). When comparing Met versus C, 519 RBPs were differentially expressed (with 70 RBPs downregulated and 449 upregulated), while Met versus Loc comparison, 505 RBPs were differentially expressed (with 97 RBPs downregulated and 408 upregulated). The most common change was increased RBP expression in metastatic prostate cancer compared to the normal tissue or early stages of cancer. In Loc versus C comparison, 157 RBPs were differentially expressed in this dataset (with 28 RBPs downregulated and 129 upregulated). Volcano plots of each comparison is shown in Figure 3H–J. The analysis of the Iglesias‐Gato dataset suggests that expression of RBPs is similar between benign prostate tissue and local, primary prostate cancer, but changes in RBP expression occur as prostate cancer progresses to metastatic form. It is noteworthy that the metastatic samples do not differ from each other much in their RBP expression patterns, indicating a level of homogeneity in the RBP expression in metastatic prostate cancer.

### 3.4 Prostate cancer progression involves significant changes in RBP phosphorylation

**FIGURE 4:**
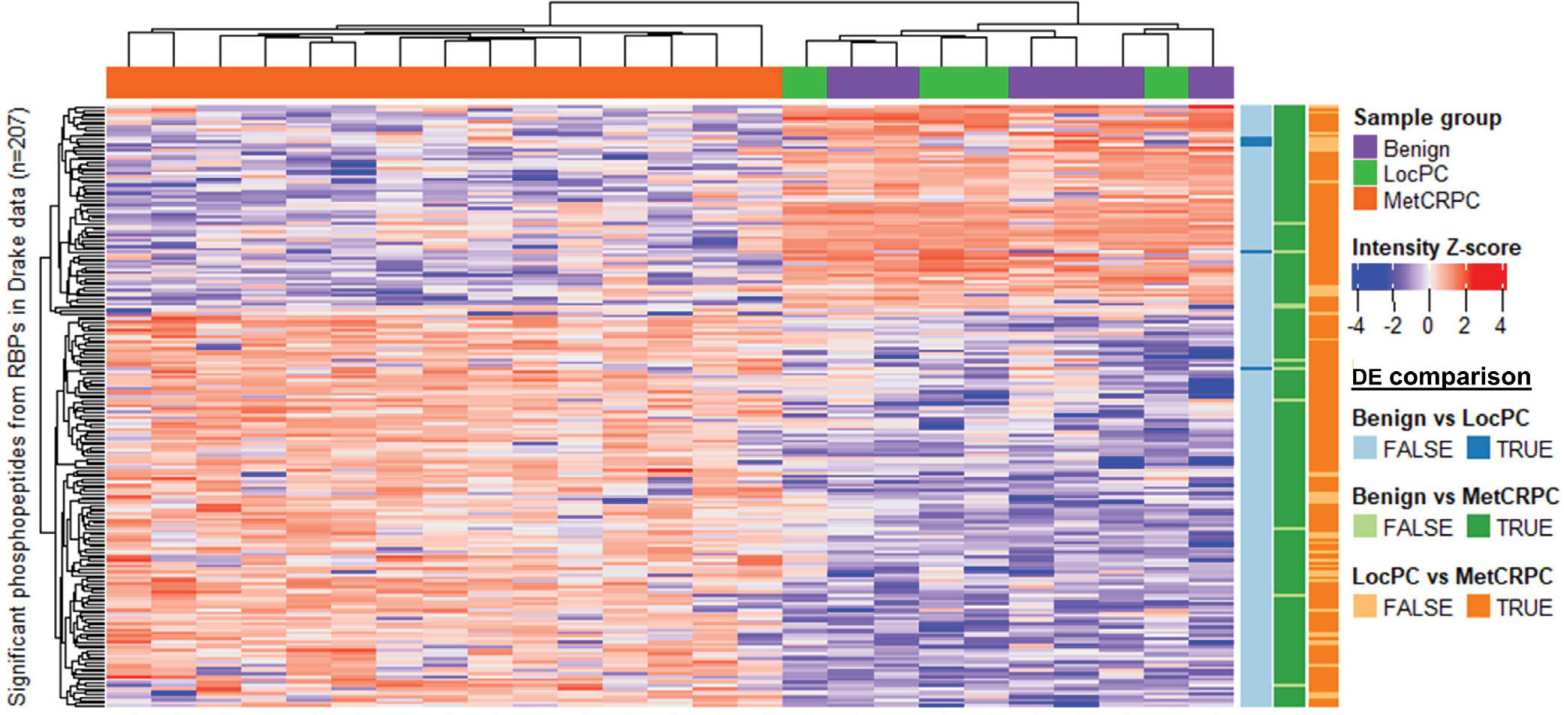
RBP phosphopeptides differentially present (DE) between local and metastatic prostate cancer. Clustering analysis of 207 phosphopeptides from 133 RBPs based on the Drake dataset. Phosphorylation patterns appear similar between benign and local prostate cancer (LocPC) samples but change as cancer progresses to metastatic CRPC (MetCRPC).

Next, we tested whether phosphorylation of RBPs changes during prostate cancer development and evolution by comparing benign, LocPC, and MetCRPC samples in the Drake dataset. In total, 207 phosphopeptides originating from 133 RBPs were differentially present in any of the comparisons (Figure 4). Hierarchical clustering based on the differential phosphopeptides divided the samples into two clear clusters: one containing all MetCRPC samples and the other containing benign and LocPC samples (Figure 4). The phosphorylation of RBPs was highly similar in benign and LocPC samples with only five phosphopeptides differentially present. In contrast, MetCRPC samples differ greatly from the other two sample groups with 197 phosphopeptides differentially present between benign and MetCRPC, and 169 phosphopeptides between LocPC and MetCRPC samples. The phosphorylation decreases in approximately one third and increases in two thirds of the cases from the early stages of PC to MetCRPC.

### 3.5 Distinct groups of RBPs are differentially expressed and phosphorylated between primary and advanced prostate cancer

**FIGURE 5:**
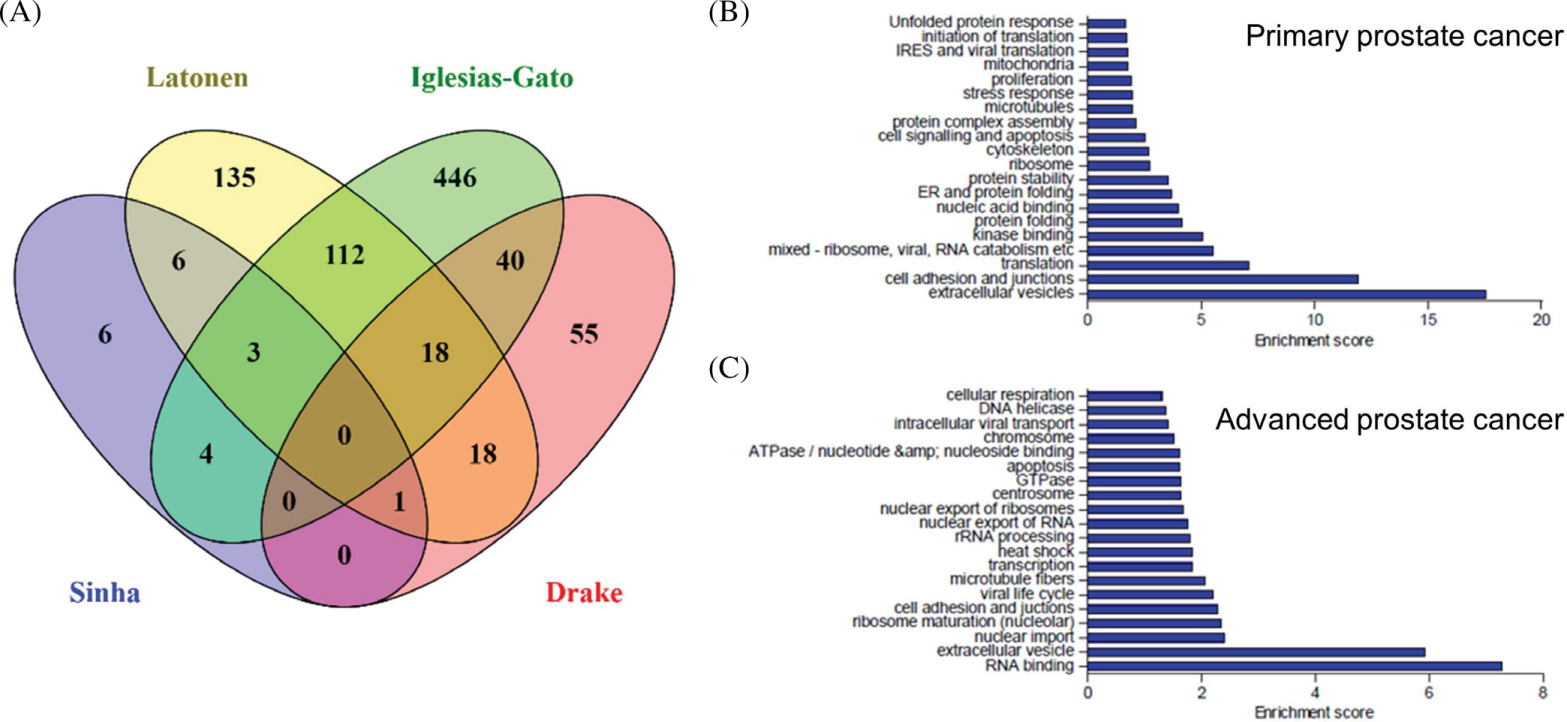
RBPs differentially expressed in prostate cancer are enriched in partly distinct pathways in early and advanced prostate cancer. (A) Venn diagram showing overlap of differentially ex‐pressed RBPs or RBP phosphopeptides between the proteomics datasets analyzed. Pathway enrichment analysis of RBPs changed (B) in the primary prostate cancer and (C) in the advanced prostate cancer based on combined data from the Latonen and the Iglesias‐Gato datasets.

We next investigated which of the differentially expressed and phosphorylated RBPs (Supplementary Table 3) are common between the studied datasets (Figure 5A). Considering that the datasets shared significant overlap in expressed RBPs (Figure 1), the number of differentially expressed RBPs that are common between the datasets is surprisingly low. This is evident particularly in curable primary prostate cancer (between Drake and Sinha datasets; Figure 5) where the differential phosphorylation events are largely separate from the differentially expressed RBPs. The differentially expressed RBPs in datasets containing both primary and advanced prostate cancer samples showed greater overlap (Latonen and Iglesias‐Gato datasets; Figure 5).

We then asked which biological functions the differentially expressed RBPs are involved in. We did pathway analyzes of RBPs that are altered in primary prostate cancer (from the Latonen dataset the comparison of PC vs. BPH, and from the Iglesias‐Gato dataset Loc vs. C, combined list including 325 RBPs) and in advanced prostate cancer (from the Latonen dataset the comparison of CRPC vs. PC, and from the Iglesias‐Gato dataset the Met vs. Loc, combined list including 605 RBPs) (Figure 5B,C; Supplementary Tables 4 and 5). The pathways enriched in both primary and advanced prostate cancer were extracellular vesicles, as well as cell adhesion and junctions (Figure 5B,C). These were the highest enriched groups in primary cancer compared to control samples, but also scored high in advanced cancer compared to primary disease (Supplementary Figure 2). In the primary cancer analysis, enriched RBP functions included translation and ribosome‐related groups, kinase binding, protein folding and stability, nucleic acid and protein binding, cytoskeleton, cell signaling, and apoptosis (Figure 5B). Of evidently cancer‐related functions, proliferation was also amongst the top 20 enriched pathways. In advanced cancer, RNA binding appeared as the most enriched function (Supplementary Figure 3). Distinct from primary cancer, the top 20 enriched pathway functions for RBPs in advanced prostate cancer included nuclear import and export pathways (Supplementary Figure 4), rRNA processing, ribosome maturation, and ribosome export. These results indicate that increased ribosome production is a central function of differentially expressed RBPs in advanced prostate cancer. Interestingly, chromatin‐related functions including transcription were also enriched in advanced disease. Further, GTPase, ATPase, and nucleotide binding, as well as DNA helicase functions are also present in the top 20 pathways. While in primary cancer, pathways for stress response and unfolded protein response are enriched, in advanced prostate cancer the heat shock response is enriched. This may indicate a different RBP‐mediated program of primary and advanced prostate cancer to respond to stress. These results highlight that the functions driven by RBPs are altered when primary prostate cancer evolves to treatment resistant and metastatic cancer.

### 3.6 Differentially expressed RBPs affect proliferation, apoptosis, and migration of prostate cancer cells

As RBP functions may contribute to drug resistance of prostate cancer, we were interested in finding RBPs that are significantly altered during disease progression. To rely on reproducible results by mass spectrometry proteomics from several sources, we focused on 66 RBPs that exhibited differential expression in more than one comparison (CRPC vs. PC in the Latonen dataset, MET vs. Loc in the Iglesias‐Gato dataset, and primary cancers with or without BCR in the Sinha dataset). LARP4 was the only RBP appearing in all three comparisons. Interestingly, LARP4 has previously been shown to regulate migration, invasion, and shape of prostate cancer cells,^28^ validating our analysis approach for identifying functionally meaningful RBPs. To find novel factors amongst the top differentially expressed RBPs that could functionally contribute to prostate cancer progression, we identified candidate RBPs that are functionally uncharacterized for their role in prostate cancer. First, we shortlisted RBPs based on the availability of prior information in published literature by prioritizing RBPs with little or no functional evidence for relevance in cancer, especially prostate cancer. We then identified the candidates that are expressed in prostate epithelium and/or prostate cancer cells based on available immunohistochemistry data in Human Protein Atlas. Finally, the selected RBPs needed to have relevant expression in prostate cancer cell lines LNCaP, PC‐3, and/or 22Rv1 according to RNA expression data by Prensner et al.^29^ (Supplementary Figure 5) to ensure that the RBPs are suitable for downregulation experiments in cell culture‐based assays. With these criteria, we selected 12 RBPs (Supplementary Table 1) to be assessed by siRNA screening for their ability to contribute to cancer‐relevant cellular functions, namely cell growth, migration, and apoptosis.

**FIGURE 6:**
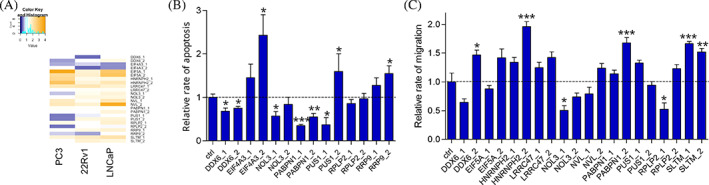
Cellular effects of siRNA targeting of RBPs in prostate cancer cells. Selected RBPs were targeted with two siRNA sequences each. (A) Relative cell growth shown as a heatmap at 6 days timepoint in the indicated cell lines. Treatments with decreased (blue) and increased (orange) growth compared to control siRNA‐treated cells (with value 1, in white) are shown. The color key demonstrates number of treatments with certain fold changes (cyan). (B) Relative rate of apoptosis in PC‐3 cells shown for siRNAs that inhibited cell growth in each cell line in A. (C) Relative rate of migration in PC‐3 cells. Error bars, SEM. *p < .05, **p < .01, ***p < .001.

We performed live cell imaging‐based assays with two siRNA sequences targeting each RBP (Supplementary Table 1). First, we assessed cell growth with LNCaP, PC‐3, and 22Rv1 cells (Figure 6A, Supplementary Figure 6). In this assay, siRNA targeting of EIF5A, HNRNPH2, LRRC47, NVL, and SLTM induced cell growth in all tested cell lines, while siPABPN1 had a mild inducer effect. Targeting EIF4A3 was the only hit in the screen with a clear growth inhibitory effect in all cell lines. siRNA targeting of DDX6, NOL3, PUS1, and RRP9 were growth‐inhibitory in PC‐3 cells while in LNCaP cells a growth‐promoting effect was observed. In 22Rv1 cells, siRNA targeting of DDX6 or RRP9 was growth‐inhibitory, while siNOL3 was growth‐promoting (Figure 6A). The siRNAs that caused cell proliferation to decrease were further studied for their effects on apoptotic rate of the cells (Figure 6B, Supplementary Figure 7). siRNA targeting of EIF4A3 significantly induced apoptosis in all three cell lines with either or both tested siRNA sequences. siDDX6 decreased apoptosis both in PC‐3 and 22Rv1 cell lines, and siPABPN1 in PC‐3 cells (Figure 6B, Supplementary Figure 7). We then tested effects of RBP siRNA targeting on the ability of prostate cancer cells to migrate (Figure 6C). We used PC‐3 cell line due to measurable migration activity of these cells in culture conditions. Increased migration was detected with siRNAs targeting of HNRNPH2, PABPN1, and SLTM. Migration ability of PC‐3 cells was clearly decreased with siRNAs targeting of NOL3 (Figure 6C, Supplementary Figure 8). These results indicate several RBPs that have functional effects relevant for cell growth, migration, and apoptosis of prostate cancer cells.

**FIGURE 7:**
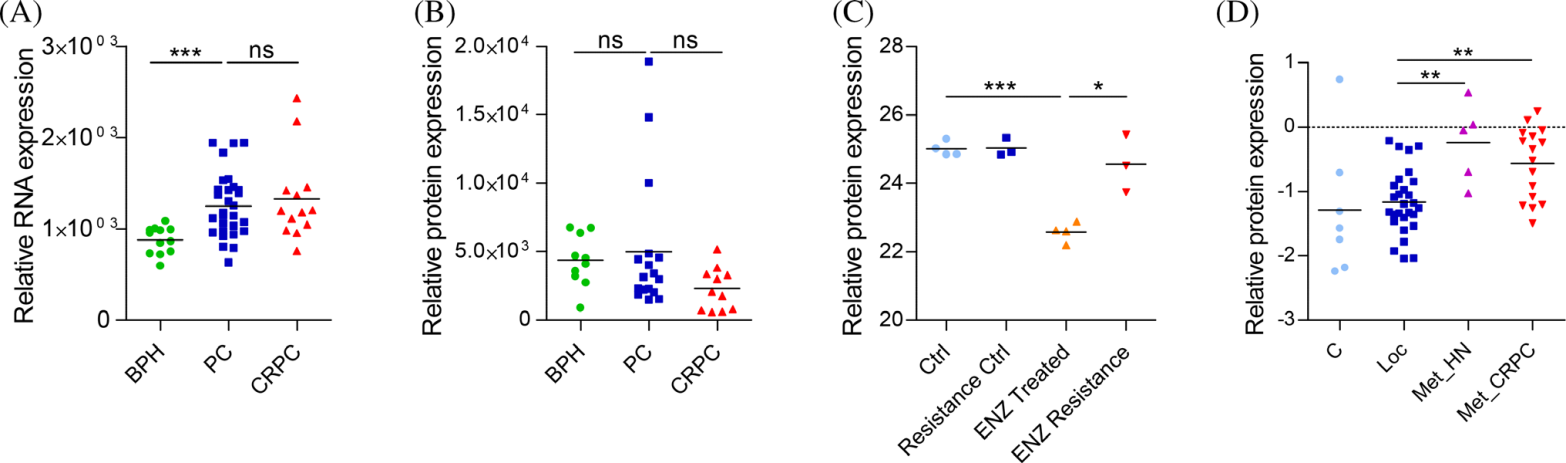
RRP9 protein levels are regulated during castration resistance and upregulated in distal metastasis in prostate cancer. RNA (A) and protein (B) expression levels of RRP9 in Tampere prostate tumor dataset according to data published in Reference 23 and the Latonen dataset^9^ (C) Protein expression levels RRP9 in LNCaP cells treated with enzalutamide for 48 h or after acquiring enzalutamide resistance according to data published in Reference 24. (D) Protein expression levels of RRP9 in local, hormone‐naïve and distal CRPC metastases according to the Iglesias‐Gato dataset^19^ Error bars, SEM. *p < .05, **p < .01, ***p < .001. ns, not significant.

We then interrogated whether these RBPs are altered in prostate cancer also at the RNA level. We queried the RNA sequencing data from the same tumors as in the Latonen proteomics dataset^30^ (Supplementary Figure 9), which showed that most RBPs (EIF4A3, EIF5A, HNRNPH2, LRRC47, PABPN1, and SLTM) exhibit similar relationships between benign, primary, and castration‐resistant disease in RNA and protein data, and they are therefore likely regulated primarily at the transcriptional level. DDX6, NOL3, and RRP9 showed differential expression in RNA and protein data between primary cancer and CRPC, suggesting that these RBPs are regulated post‐transcriptionally in prostate cancer. Interestingly, in CRPC tumors the protein expression of RRP9 is downregulated although RNA expression stays at a comparable level to primary tumors (Figure 7A,B). This indicates that RRP9 is downregulated in local CRPC tumors post‐transcriptionally. Silencing of RRP9 (Supplementary Figure 10) induced apoptosis in prostate cancer cells (see Figure 6B), suggesting that RRP9 may support cancer cell survival. Thus, we interrogated RRP9 levels protein levels after androgen deprivation treatment with enzalutamide, a common ADT drug currently in clinical use, based on data published in Blomme et al.^31^ In LNCaP cells treated with 10 μM enzalutamide for 48 h, RRP9 levels are significantly decreased (Figure 7C). However, the levels are recovered in cells that have acquired resistance to the treatment. Furthermore, RRP9 levels are increased in distal metastasis both in hormone‐naïve and castration‐resistant samples (Figure 7D). Collectively, these results suggest that recovery from treatment‐induced downregulation of RRP9 is associated with treatment resistance, and that RRP9 protein is upregulated in metastases in prostate cancer.

## 4 DISCUSSION

As RNA regulation and RBPs have been understudied for their alterations and roles in prostate cancer, we performed systematic assessment of RBP expression along the different stages of prostate cancer. Recent advancements in proteomics methods have enabled quantitative assessment of protein expression changes in a large scale in diseased tissues. This has revealed that, compared to DNA and RNA sequencing data only, additional altered mechanisms and pathways are found from cancer, and that certain frequent and systematic changes are detectable only at the protein level.^6^, ^7^, ^8^, ^9^, ^10^, ^11^, ^12^ Hence, proteome‐wide knowledge of differentially regulated proteins is valuable for understanding tumor evolution and for developing better cancer treatments.

By integrating genome‐wide genetic, transcriptomic, and proteomic data from the same tumors, we recently found that prostate cancer harbors changes in several cellular pathways governed by RBPs, and that many of these alterations are detected mainly at the protein level.^9^ This prompted us to investigate the proteomic changes in RBPs in prostate cancer in more detail. We analyzed the available proteome‐wide datasets to analyze RBP profiles and to identify RBPs with significant changes in their expression and/or phosphorylation in either primary or advanced prostate cancer. We found that in primary prostate cancer, systematic alterations in RBPs are mild: there are only minor changes in the expression of RBPs between nonmalignant prostate tissue and localized prostate cancer. In contrast, several functional groups of RBPs are altered in castration resistant and metastatic prostate cancer. We identify several significant RBPs to study for their possible roles in prostate cancer development and progression. Further, we provide initial evidence in cellular assays for potential functional importance for selected RBPs.

In this work, we utilized proteomics data produced by us^9^ and others.^16^, ^17^, ^18^, ^19^ While these datasets are not exhaustive due to current technical limitations in high throughput proteomics techniques, they provide a near‐comprehensive, proteome‐wide view to prostate cancer. Hence, they jointly offer a valuable resource for assessing protein profiles in prostate cancer that are not visible through DNA or RNA sequencing studies. RBPs are not frequently mutated in cancer, and their expression changes are not always evident from their mRNA levels.^32^ Yet, our analyzes here show that RBP profiles are significantly changed in prostate cancer. Hence, the posttranscriptional molecular mechanisms that are responsible for the differential expression and posttranslational modifications of key RBPs in prostate cancer are of interest for future studies.

Although RBPs were generally uniformly present throughout the datasets, our analysis revealed that different stages of prostate cancer involve distinct alterations in RBP expression and posttranslational modifications by phosphorylation. While the core functions of RBPs in poly(A)RNA regulation and ribosome production are known to be important for cancer and are showcased also in this study, our analyzes reveal further interesting aspects of RBP roles in prostate cancer. The enrichment of extracellular vesicle functions in both primary and advanced disease may indicate that RBPs contribute to active vesicle secretion by prostate cancer cells. Hence, RBP alterations may be of relevance when extracellular vesicles are utilized with liquid biopsies. The elevated transcription‐, chromosome‐, and DNA helicase‐associated RBP functions in advanced disease suggest increased chromatin‐associated roles of RBPs in CRPC and metastatic disease. Interestingly, several RBPs are known to bind DNA in addition to RNA, and to have roles in regulating transcription.^33^ These RBPs include e.g. FUS which interacts with AR,^34^, ^35^, ^36^ a key transcription factor driving prostate cancer. The roles of differentially expressed RBPs in regulating the transcriptional programs in advanced prostate cancer should be thus investigated. Further, the stress responsive RBPs detected to be enriched in our pathway analysis could contribute to drug responses and formation of drug resistance in prostate cancer.

The pools of identified RBPs in each disease stage warrant further investigation for possible functional roles in disease development and progression. In primary cancer, KIF1C (Kinesin family member 1C) was differentially expressed according to T‐stage in primary prostate cancer. KIF1C regulation has recently been associated with invadopodia,^37^ suggesting that KIF1C may be associated with prostate tumor invasion and metastasis. Three RBPs were differentially expressed in primary tumors harboring ETS gene fusions, namely ALDH6A1, SPATS2L, and LRP1. ALDH6A1 is a mitochondrial aldehyde dehydrogenase which has been associated with drug metabolism in cancer.^38^ While expression of LRP1 (low‐density lipoprotein receptor‐related protein 1) is known to be decreased in prostate cancer^39^ and may be mostly expressed in prostate stroma, SPATS2L (Spermatogenesis associated serine rich 2 like) is a functionally uncharacterized stress granule and nucleolar protein that is expressed in tumor cells and warrants further investigation. Most ETS gene fusions in prostate cancer bring the gene that is coding for ERG under a regulatory sequence controlled by androgens, resulting in de novo expression of this oncogenic transcription factor in prostate cancer cells.^40^ It remains to be investigated whether the RBPs identified here to be differentially expressed according to the ETS status of the tumors are transcriptional targets of ERG. The only RBP that was identified to be associated with advancement of prostate cancer based on all three nonphosphoproteomic datasets was LARP4 (La ribonucleoprotein 4). LARP4 belongs to an evolutionarily conserved family of La‐related proteins regulating transcription and translation.^41^ LARP4 can bind poly(A) mRNAs and promote mRNA stability,^42^ and it was previously identified in an RNAi screen as a gene that regulates migration, invasion, and shape of PC‐3 cells.^26^

We screened cellular effects of shortlisted RBP candidates that we identified to be 1) differentially expressed in prostate cancer and 2) expressed sufficiently in prostate cancer cell lines to be qualified for siRNA targeting. We identified several RBPs with indicated functions in cell growth, apoptosis, and migration. siRNA targeting of EIF5A and HNRNPH2 both increased cell growth and migration when downregulated, indicating that these proteins may have important gatekeeper functions for cell division and motility. HNRNPH2 (Heterogeneous nuclear ribonucleoprotein H2) is a component of heterogeneous nuclear ribonucleoprotein (hnRNP) complex that is associated with neuropathological conditions. HNRNPH2 functions are uncharacterized in cancer. eIF5A is a translation initiation factor that functions in translation elongation and is implicated in transcription, mRNA turnover, and nucleocytoplasmic transport.^43^ Elevated expression of eIF5A is associated with unfavorable prognosis in several cancers, but direct evidence for a role for eIF5A in prostate cancer is lacking. EIF4A3, another translation initiation factor, was the only RBP in the siRNA screen for which a clear growth inhibitory effect was indicated in all cell lines and targeting of which also induced apoptosis. These results suggest that the function of EIF4A3 is essential for prostate cancer cells. EIF4A3 is involved in RNA splicing and nonsense‐mediated mRNA decay,^44^ and functions for it in cell cycle and RNA stress granule formation have been indicated.^45^ Overexpression of EIF4A3 mRNA is linked to poor prognosis in breast and lung cancer,^46^ and EIF4A3‐selective inhibitors have been studied as potential cancer therapy with promising results in xenograft mice.^47^ Collectively, these results suggest that functions and targeting of EIF4A3 should be further explored as a potential therapeutic avenue in prostate cancer.

The data presented here indicates that RRP9 is regulated post‐transcriptionally in cells during treatment responses and castration resistance. Further, increased RRP9 protein expression is associated with metastasis in prostate cancer. RRP9 is a U3 small nucleolar RNA binding protein essential for 18S rRNA processing and small subunit ribosome formation.^48^ Previously, elevated RRP9 expression has been reporter in colorectal cancer, and downregulation of RRP9 in colon cancer cells inhibits subcutaneous tumor formation in xenografted mice.^49^ Further, RRP9 promotes gemcitabine resistance in pancreatic cancer through activating AKT signaling pathway.^50^ Collectively, these results suggest that RRP9 has tumor‐promoting functions in cancer, and that the utility of it in preventing prostate cancer metastasis should be explored.

Our work demonstrated that the RBP profiles in prostate tissue change as prostate cancer develops, forms drug resistance, and progresses to metastasize. Our work identified a panel of RBPs that are significantly altered for their presence in prostate cancer cells, and we indicated functional significance for several of them. Further investigation is warranted to understand the detailed roles of these factors in prostate cancer, the molecular mechanisms behind their alterations, and their potential utility in future cancer targeting.

## AUTHOR CONTRIBUTIONS

**Erika Aikio:** Data curation (equal); formal analysis (equal); investigation (equal); visualization (equal); writing – original draft (equal). **Sonja Koivukoski:** Conceptualization (supporting); methodology (supporting); supervision (supporting); validation (equal); writing – review and editing (supporting). **Elina Kallio:** Formal analysis (supporting); investigation (supporting); validation (supporting); visualization (supporting); writing – original draft (supporting); writing – review and editing (supporting). **Nithin Sadeesh:** Data curation (supporting); investigation (supporting); writing – review and editing (supporting). **Einari Niskanen:** Data curation (equal); formal analysis (supporting); methodology (equal); supervision (equal); visualization (supporting); writing – review and editing (equal). **Leena Latonen:** Conceptualization (lead); formal analysis (supporting); funding acquisition (lead); investigation (supporting); methodology (supporting); project administration (lead); resources (lead); software (supporting); supervision (equal); validation (supporting); visualization (equal); writing – original draft (equal); writing – review and editing (lead).

## CONFLICT OF INTEREST STATEMENT

The author declares there is no potential conflict of interest.

## ETHICS STATEMENT

Ethics approval and consent to participate are not applicable to this study.

## Supporting information


**Supplementary Figure 1** Expression of RBPs in the proteomics datasets used in the study. Heatmaps shown for each dataset with clustering based on RBP expression levels in each sample. Number of RBPs being present is 792 for Latonen dataset^5^ (A), 1234 RBPs after filtering for Iglesias‐Gato dataset^15^ (B), 1236 RBPs after filtering for Sinha dataset^13^ (C), and 1813 phosphopeptides originating from 742 RBPs for Drake dataset^16^ (D).
**Supplementary Figure 2.** Expression of RBPs involved in adherens junctions is altered during prostate cancer development and progression. Heatmaps showing RBPs involved in adherens junctions in Latonen dataset^5^ (A) and in Iglesias‐Gato dataset^15^ (B) datasets.
Supplementary Figure 3. Expression RBPs involved in poly(A) RNA binding is altered during prostate cancer development and progression. Heatmaps showing RBPs involved in poly(A) RNA binding in Latonen dataset^5^ (A) and in Iglesias‐Gato dataset^15^ (B) datasets.
**Supplementary Figure 4.** The expression of RBPs involved in nucleocytoplasmic transport is altered during prostate cancer development and progression. Heatmaps showing RBPs involved in nucleocytoplasmic transport in Latonen dataset^5^ (A) and in Iglesias‐Gato dataset^15^ (B) datasets.
**Supplementary Figure 5.** RBP expression in prostate cancer cells. Levels of RNA expression of the selected RBPs in PC‐3, 22Rv1, and LNCaP prostate cancer cells according to dataset by Prensner et al.^29^
**Supplementary Figure 6.** Effects of siRNA downregulation of RBPs on growth rate of prostate cancer cells. Example images of IncuCyte cell images for each siRNA in (A) PC‐3, (B) 22Rv1, and (C) LNCaP cells.
**Supplementary Figure 7.** Effects of siRNA downregulation of RBPs on apoptotic rate of prostate cancer cells. Selected RBPs were targeted with two siRNA sequences each. LNCaP (A), and 22Rv1 (B) shown for siRNAs that inhibited cell growth in each cell line in Figure 6A. (C) Example images of IncuCyte apoptotic cell detection for each siRNA in each cell line used. Error bars, SEM. *p < .05, **p < .01, ***p < .001.
**Supplementary Figure 8.** Effects of siRNA downregulation of RBPs on migration rate of PC‐3 prostate cancer cells. Example images of IncuCyte migration detection images detection for each siRNA.
**Supplementary Figure 9.** RBP expression in prostate cancer. RNA and protein expression in samples of the Tampere patient cohort is shown for the indicated RBPs, RNA expression based on RNA sequencing in Ylipää et al. (23) and protein expression based on mass spectrometry proteomics in Latonen et al. (9). BPH, benign prostatic hyperplasia; PC, primary prostate cancer; CRPC, locally advanced castration resistant prostate cancer.
**Supplementary Figure 10.** RT‐qPCR from siRNA‐transfected PC‐3 cells showing downregulation of expression of RRP9. Error bars, SEM. **p < .01

**Supplementary Table 1** Expression patterns and siRNAs used for shortlisted RBPs.
**Supplementary Table 2.** RBPs present in datasets utilized in the study.
**Supplementary Table 3.** RBPs significantly differentially expressed between sample types in the datasets used utilized in this study.
**Supplementary Table 4.** The 20 annotation clusters with highest enrichment score in pathway analysis of RBPs that are altered in primary prostate cancer.
**Supplementary Table 5.** The 20 annotation clusters with highest enrichment score in pathway analysis of RBPs that are altered in advanced prostate cancer.

## Data Availability

The data that supports the findings of this study are available in the supplementary material of this article.
