# Supplementary material for: Complementary analysis of proteome‐wide proteomics reveals changes in RNA binding protein‐profiles during prostate cancer progression
Source: Cancer Rep (Hoboken). 2023 Aug 17;6(10):e1886. doi: 10.1002/cnr2.1886 (PMC10598248; doi:10.1002/cnr2.1886)
Supplement: Supplementary file 1 — Supplementary Figure 1 Expression of RBPs in the proteomics datasets used in the study. Heatmaps shown for each dataset with clustering based on RBP expression levels in each sample. Number of RBPs being present is 792 for Latonen dataset 5 (A), 1234 RBPs after filtering for Iglesias‐Gato dataset 15 (B), 1236 RBPs after filtering for Sinha dataset 13 (C), and 1813 phosphopeptides originating from 742 RBPs for Drake dataset 16 (D). Supplementary Figure 2. Expression of RBPs involved in adherens junctions is altered during prostate cancer development and progression. Heatmaps showing RBPs involved in adherens junctions in Latonen dataset 5 (A) and in Iglesias‐Gato dataset 15 (B) datasets. Supplementary Figure 3. Expression RBPs involved in poly(A) RNA binding is altered during prostate cancer development and progression. Heatmaps showing RBPs involved in poly(A) RNA binding in Latonen dataset 5 (A) and in Iglesias‐Gato dataset 15 (B) datasets. Supplementary Figure 4. The expression of RBPs involved in nucleocytoplasmic transport is altered during prostate cancer development and progression. Heatmaps showing RBPs involved in nucleocytoplasmic transport in Latonen dataset 5 (A) and in Iglesias‐Gato dataset 15 (B) datasets. Supplementary Figure 5. RBP expression in prostate cancer cells. Levels of RNA expression of the selected RBPs in PC‐3, 22Rv1, and LNCaP prostate cancer cells according to dataset by Prensner et al. 29 Supplementary Figure 6. Effects of siRNA downregulation of RBPs on growth rate of prostate cancer cells. Example images of IncuCyte cell images for each siRNA in (A) PC‐3, (B) 22Rv1, and (C) LNCaP cells. Supplementary Figure 7. Effects of siRNA downregulation of RBPs on apoptotic rate of prostate cancer cells. Selected RBPs were targeted with two siRNA sequences each. LNCaP (A), and 22Rv1 (B) shown for siRNAs that inhibited cell growth in each cell line in Figure 6A. (C) Example images of IncuCyte apoptotic cell detection for each siRNA in each cell lin [file CNR2-6-e1886-s002.pdf]

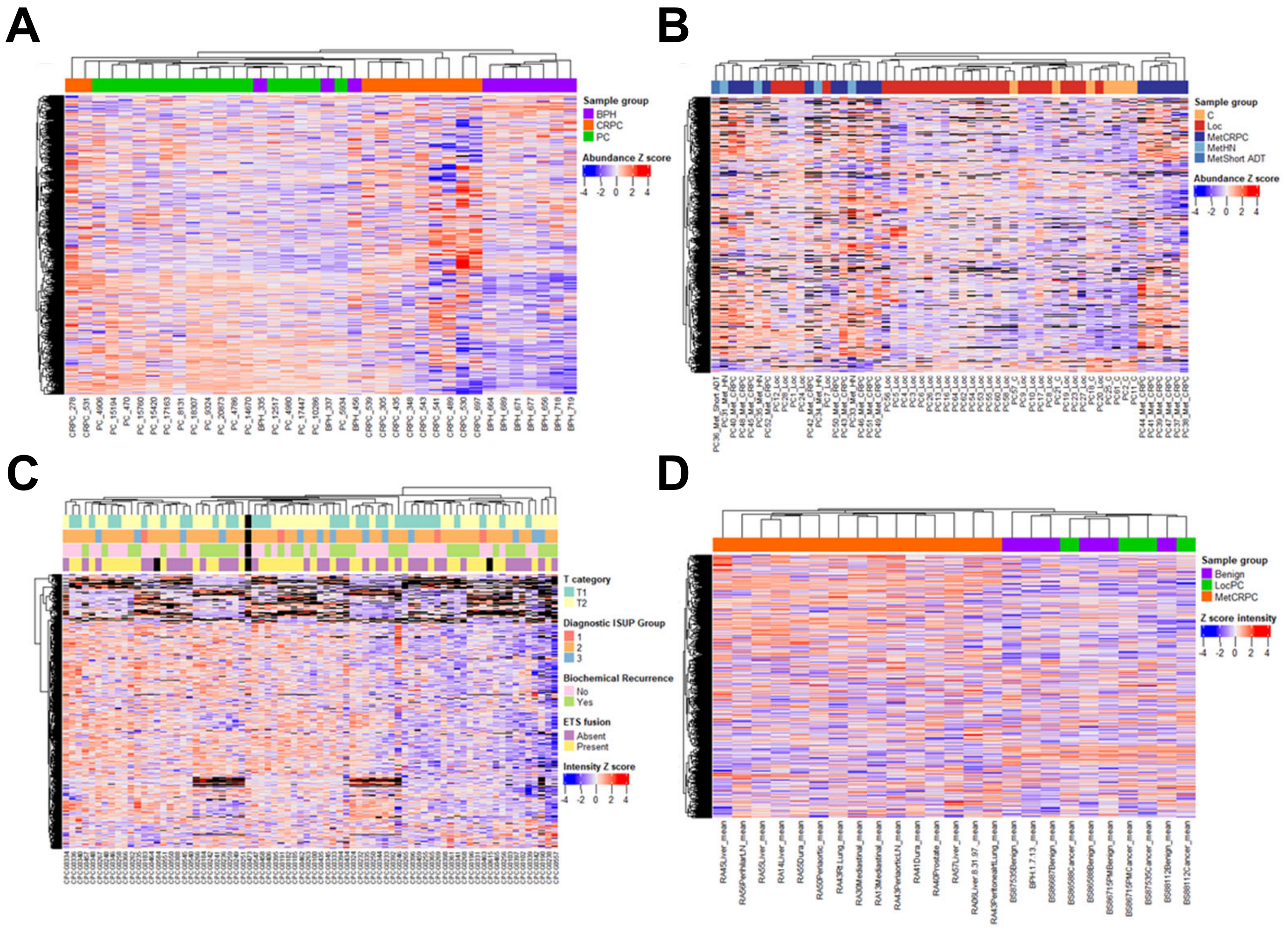

Supplementary figure 1. Expression of RBPs in the proteomics datasets used in the study. Heatmaps shown for each dataset with clustering based on RBP expression levels in each sample. Number of RBPs being present is 792 for Latonen dataset [5] (A), 1,234 RBPs after filtering for Iglesias-Gato dataset [15] (B), 1,236 RBPs after filtering for Sinha dataset [13] (C), and 1,813 phosphopeptides originating from 742 RBPs for Drake dataset [16] (D).

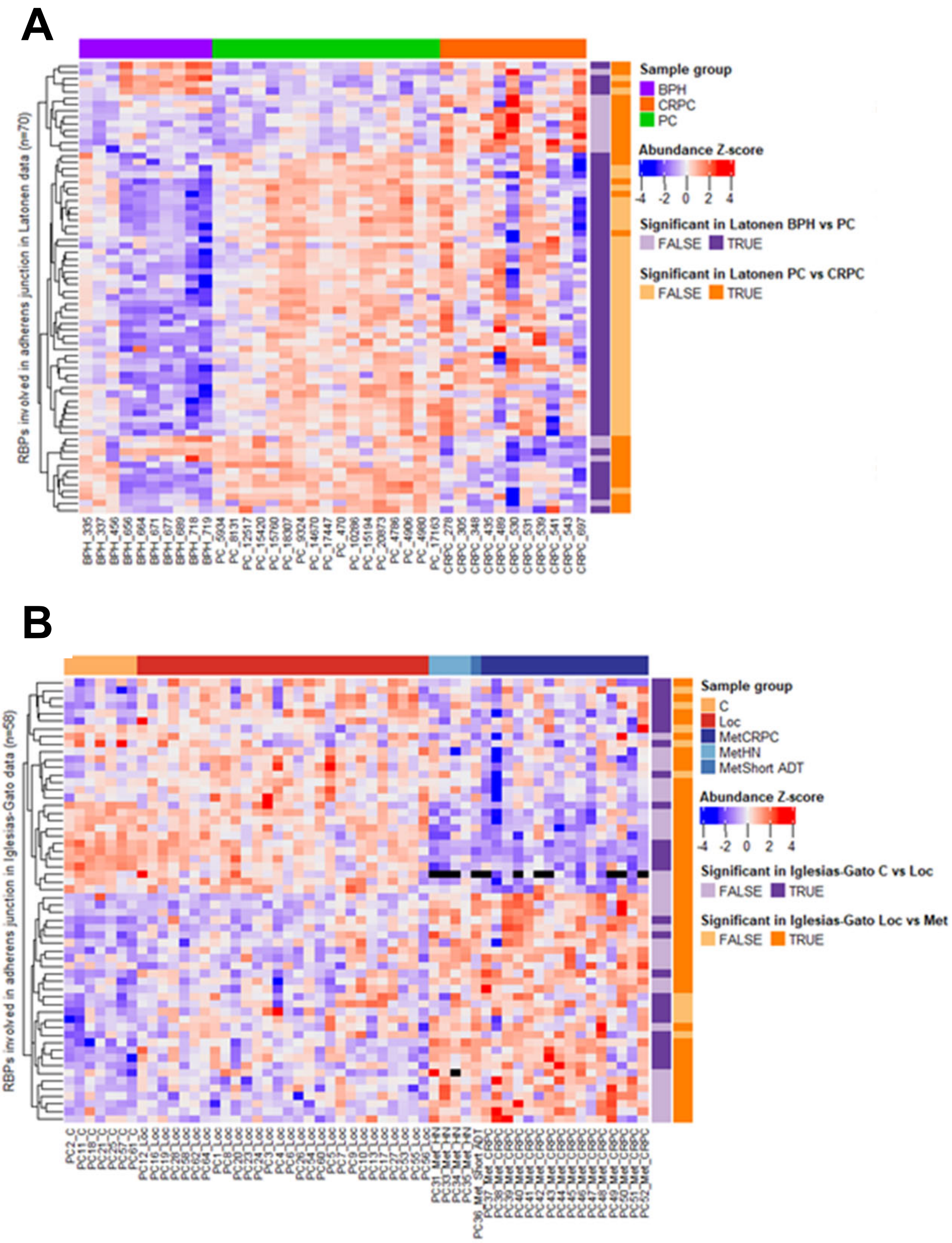

Supplementary Figure 2. Expression of RBPs involved in adherens junctions is altered during prostate cancer development and progression. Heatmaps showing RBPs involved in adherens junctions in Latonen dataset [5] (A) and in Iglesias-Gato dataset [15] (B) datasets.

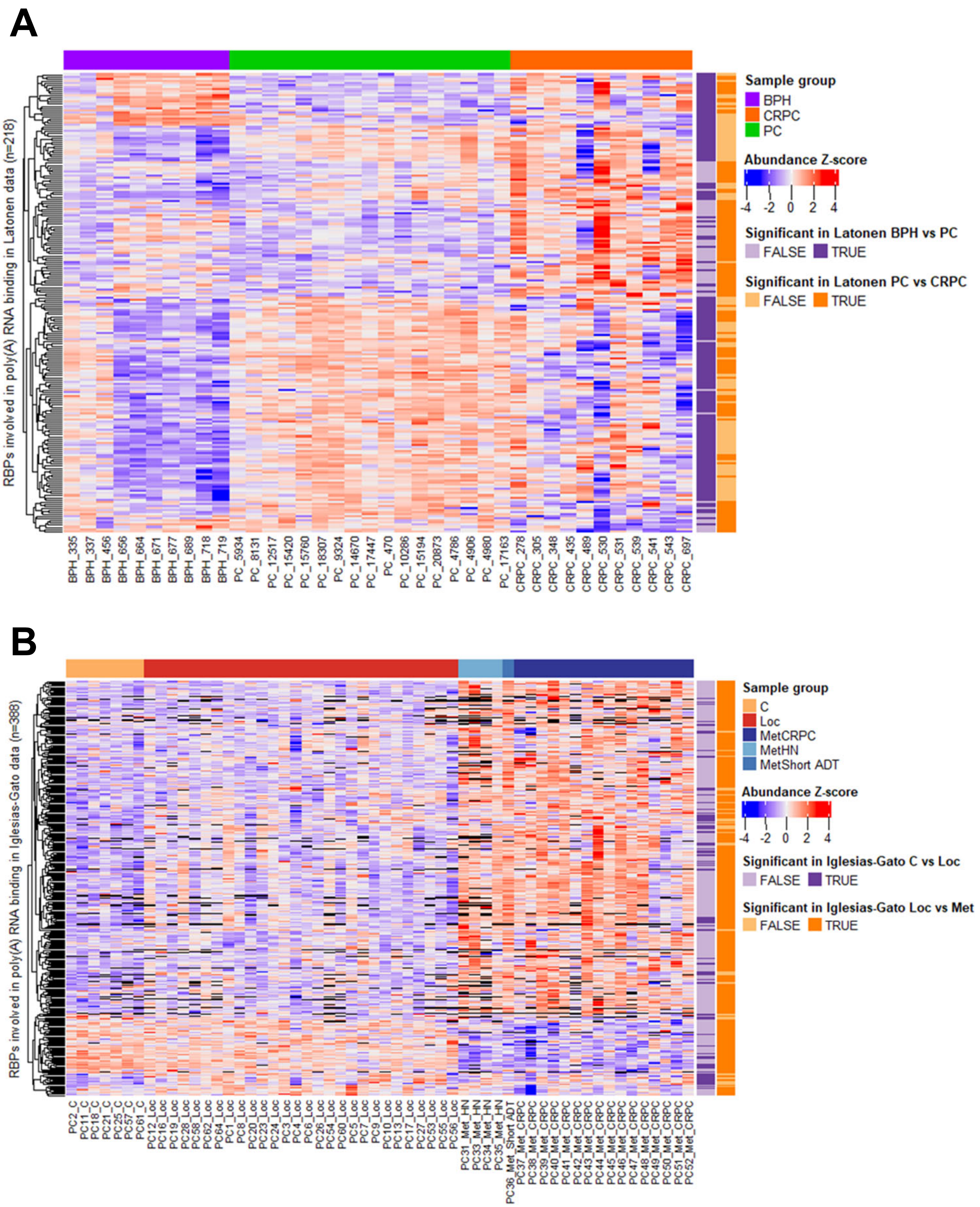

Supplemetary Figure 3. Expression RBPs involved in poly(A) RNA binding is altered during prostate cancer development and progression. Heatmaps showing RBPs involved in poly(A) RNA binding in Latonen dataset [5] (A) and in Iglesias-Gato dataset [15] (B) datasets.

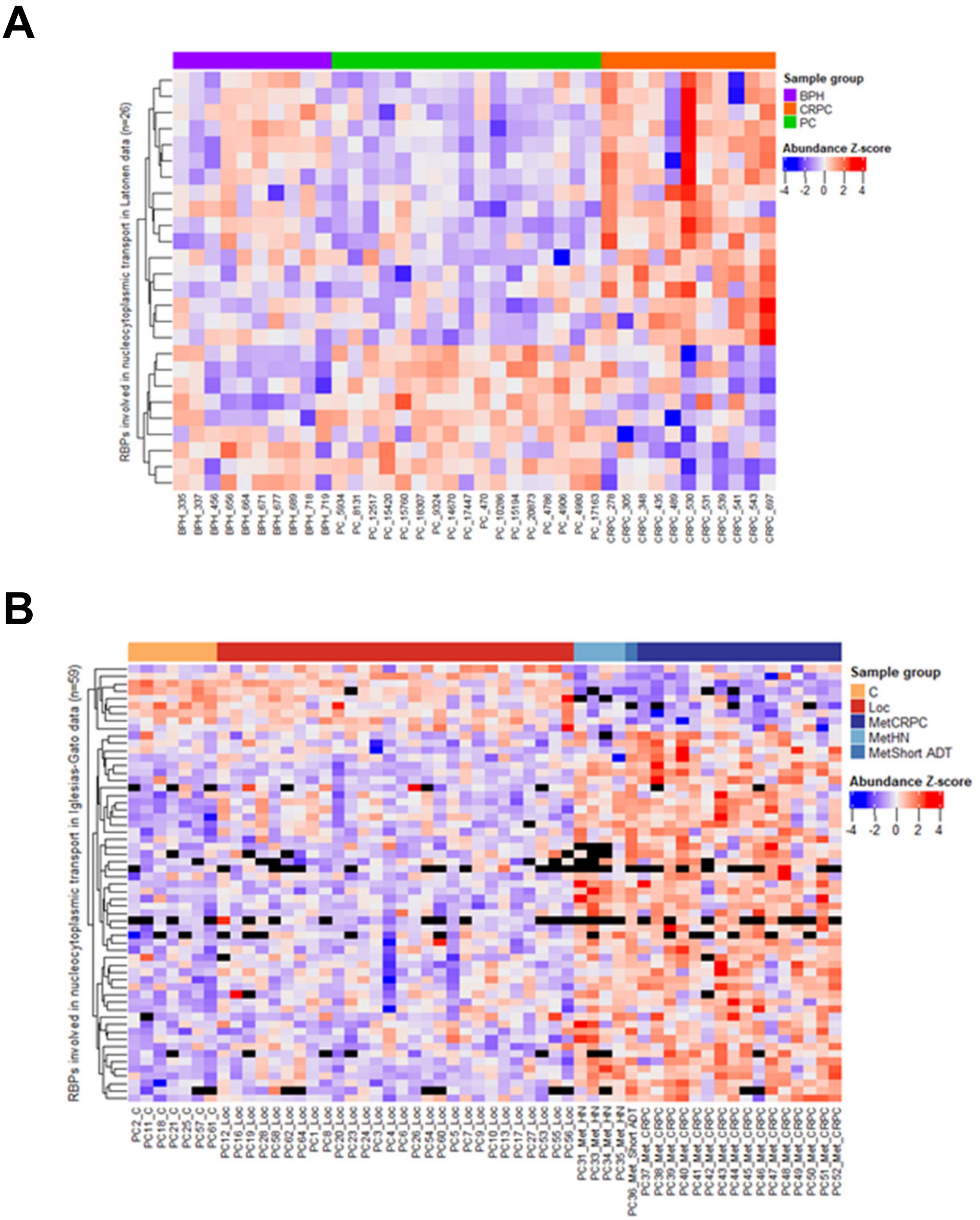

Supplementary figure 4. The expression of RBPs involved in nucleocytoplasmic transport is altered during prostate cancer development and progression. Heatmaps showing RBPs involved in nucleocytoplasmic transport in Latonen dataset [5] (A) and in Iglesias-Gato dataset [15] (B) datasets.

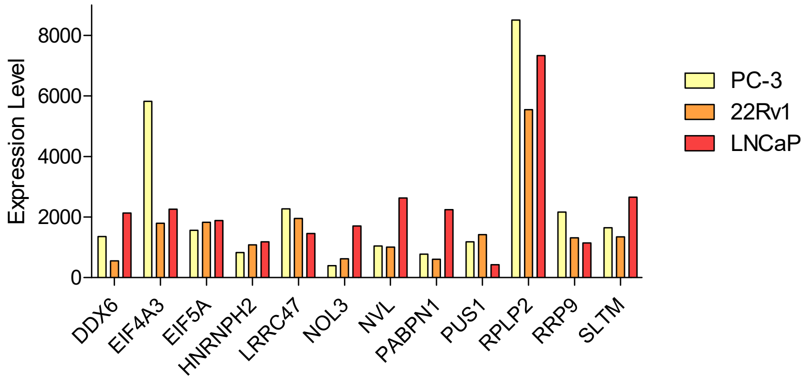

Supplementary Figure 5. **RBP expression in prostate cancer cells.** Levels of RNA expression of the selected RBPs in PC-3, 22Rv1, and LNCaP prostate cancer cells according to dataset by Prensner et al. [30].

A

B

C

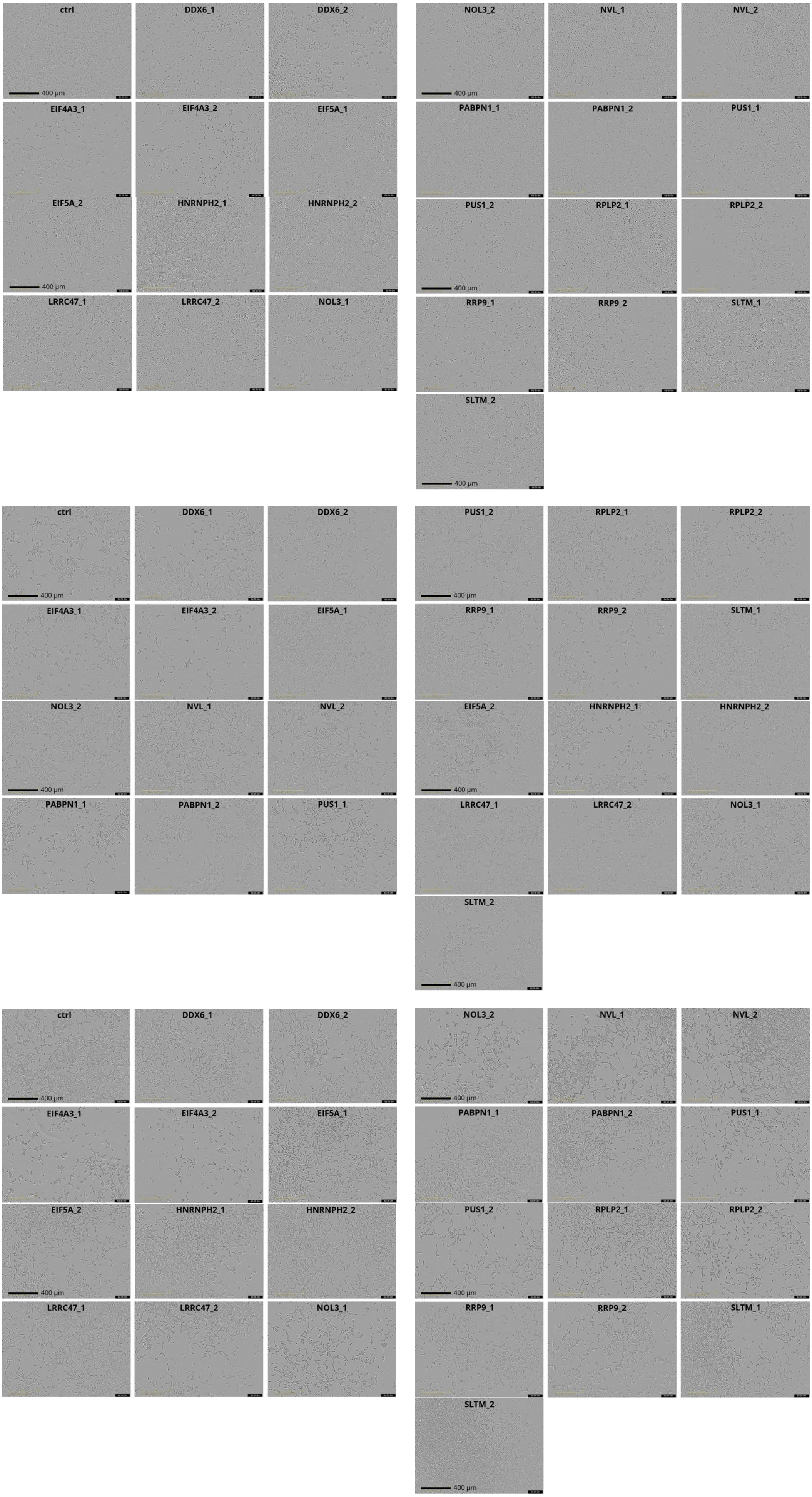

Supplementary Figure 6. **Effects of siRNA downregulation of RBPs on growth rate of prostate cancer cells.** Example images of IncuCyte cell images for each siRNA in (A) PC-3, (B) 22Rv1, and (C) LNCaP cells.

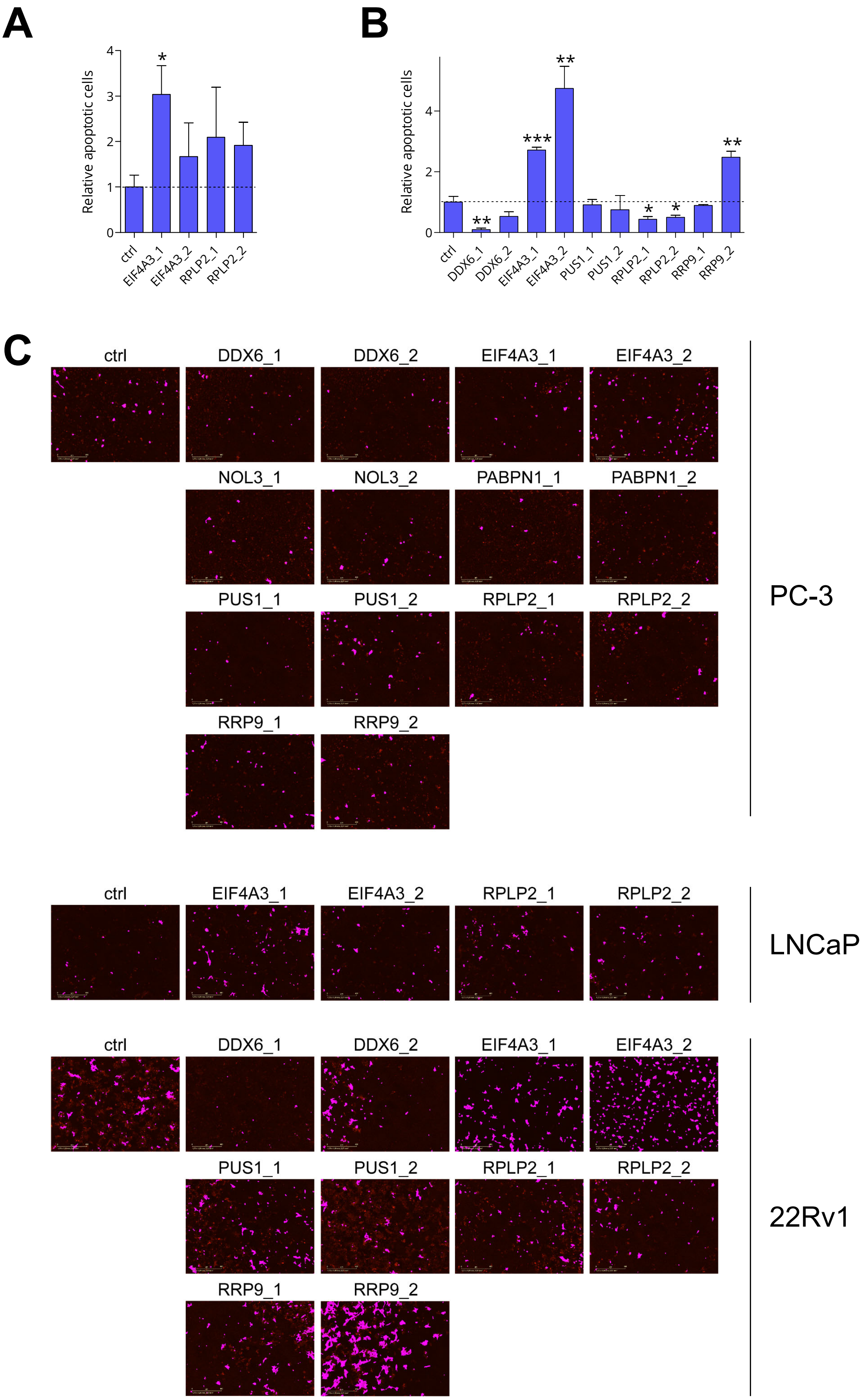

Supplementary Figure 7. Effects of siRNA downregulation of RBPs on apoptotic rate of prostate cancer cells. Selected RBPs were targeted with two siRNA sequences each. LNCaP (A), and 22Rv1 (B) shown for siRNAs that inhibited cell growth in each cell line in Figure 6A. (C) Example images of IncuCyte apoptotic cell detection for each siRNA in each cell line used. Error bars, SEM. *$p < 0.05$, **$p < 0.01$, ***$p < 0.001$.

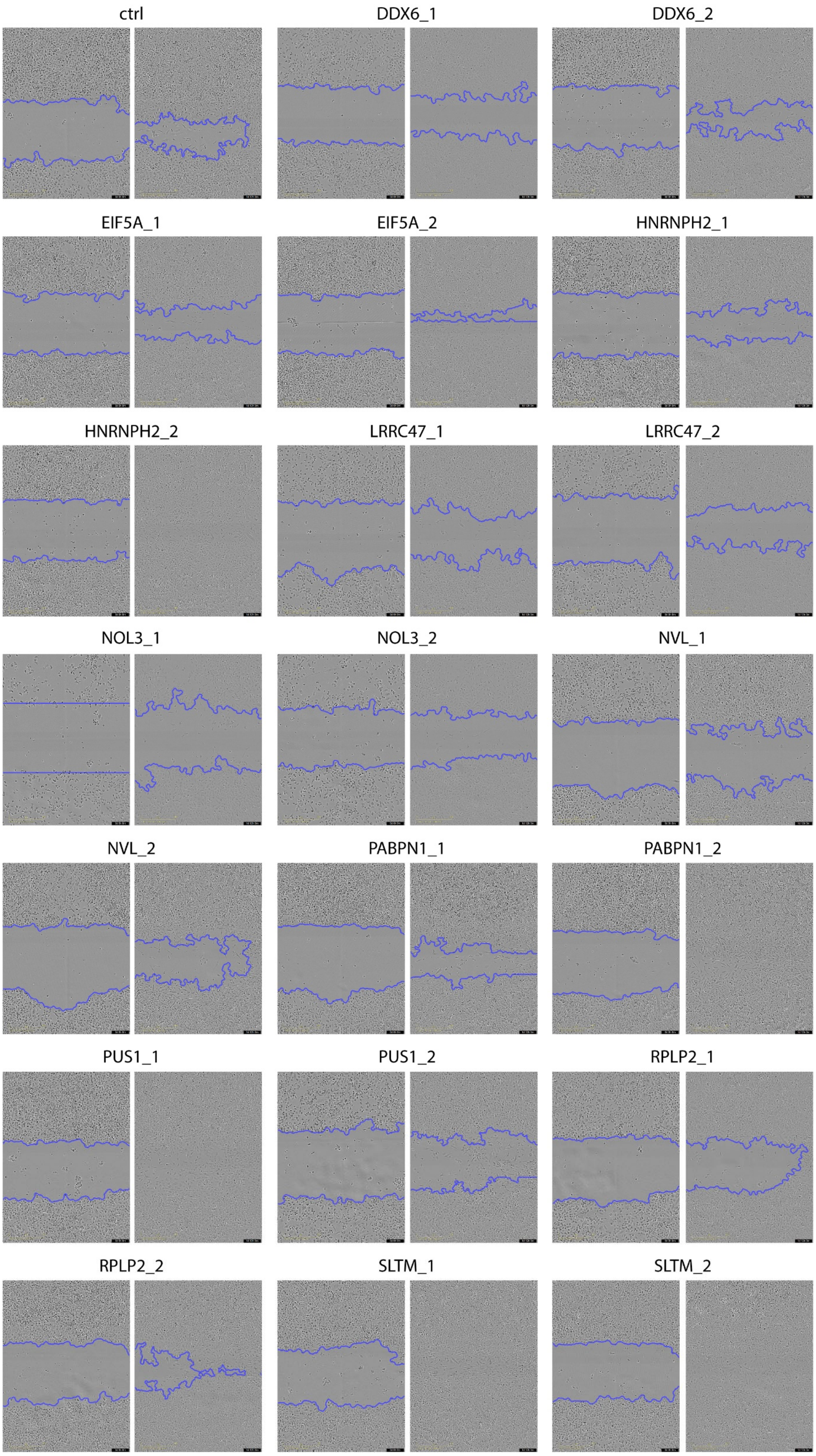

Supplementary Figure 8. **Effects of siRNA downregulation of RBPs on migration rate of PC-3 prostate cancer cells.** Example images of IncuCyte migration detection images detection for each siRNA.

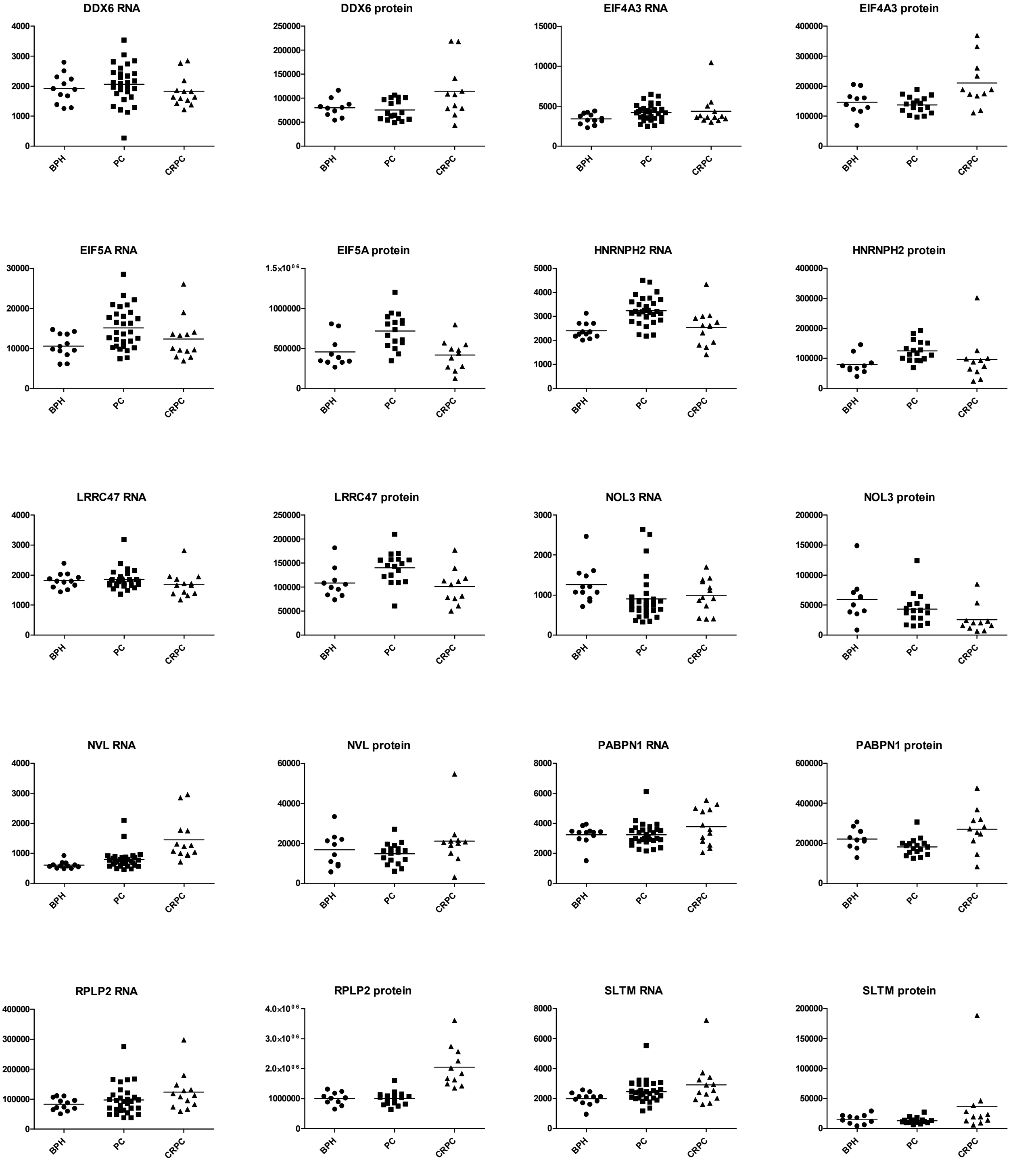

Supplementary Figure 9. **RBP expression in prostate cancer.** RNA and protein expression in samples of the Tampere patient cohort is shown for the indicated RBPs , RNA expression based on RNA sequencing in Ylipää et al. (23) and protein expression based on mass spectrometry proteomics in Latonen et al. (9). BPH, benign prostatic hyperplasia; PC, primary prostate cancer; CRPC, locally advanced castration resistant prostate cancer.

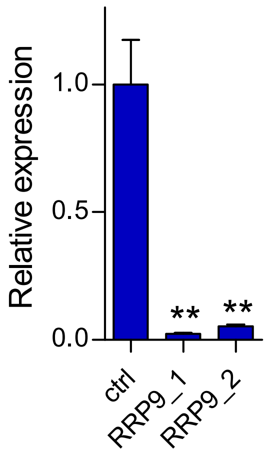

Supplementary Figure 10. **RT-qPCR from siRNA-transfected PC-3 cells showing downregulation of expression of RRP9.** Error bars, SEM. **p < 0.01.
